# Trends and Outlooks in Synthetic Biology: A Special Issue for Celebrating 10 Years of *Life* and Its Landmarks

**DOI:** 10.3390/life12020181

**Published:** 2022-01-26

**Authors:** Norizaku Ichihashi, Pasquale Stano

**Affiliations:** 1 Department of Life Science, Graduate School of Arts and Science, Komaba Institute of Science, The University of Tokyo, Tokyo 153-8505, Japan; 2 Department of Biological and Environmental Sciences and Technologies (DiSTeBA), University of Salento, 73100 Lecce, Italy

Since its inception in December 2011, Board Editors, Guest Editors, as well as the Editorial Office of *Life* have been working hard to make *Life* an outstanding journal that receives the highest-quality submissions. Important goals have been achieved, such as the inclusion of *Life* in the major databases—such as PubMed, Scopus, and the Science Citation Index Expanded (SCIE)—and especially the diffusion of a shared perception that *Life* can provide its authors with the visibility and impact that they desire. The Special Issue on “Trends and Outlooks in Synthetic Biology” was planned to celebrate the first decade of the journal and to promote *Life* as an emerging journal in the new field of synthetic biology (SB).

SB—defined as the sci-tech arena focused on (*i*) the design and construction of new biological parts, devices, and systems and (*ii*) the re-design of existing natural biological systems for useful purposes—is a young discipline, born in the early 2000s thanks to the intuition of visionary researchers, mainly in the US [1,2,3,4,5,6]. Since then, SB has attracted the attention of thousands of scientists and students (e.g., thanks to the iGEM initiative) all over the world, and today it is recognized as one of the most innovative research areas of the XXI century.

SB applies engineering approaches to biology following two complementary strategies, namely “bottom-up” and “top-down”, both providing unparallel progress in knowledge and application. The impressive biotechnological and conceptual advancements of the past two decades have led to functional tools that have empowered SB. Just to mention a few, consider the concept of “standard biological part”, or the genome editing tools such as CRISPR-Cas9, the so-called “orthogonal” biomolecules (such as the xenoforms of nucleic acids or peptides/proteins incorporating non natural amino acids), or the very idea of minimal “synthetic cells” (SCs). Notably, experimental advancements have been accompained by elegant bioinformatics and systems biology tools and models.

As Guest Editors of the 10-year celebrative Special Issue on “Trends and Outlooks in Synthetic Biology”, our efforts aimed at assembling a collection of highly relevant SB articles. Admittedly, the participation of a group of prominent Authors facilitated our efforts at a great extent.

Several Authors reported current advancements in cell-free protein synthesis (CFPS). Indeed, this is a pivotal topic in SB with multifaceted applications, either as proper tool (e.g., production of proteins of practical utility) or as core “module” in bottom-up SCs construction [7,8,9,10,11]. Kei Fujiwara and collaborators presented a research article where the complex interplay of nucleotide triphosphate (NTP) concentration, cell-extract concentration, batch/dialysis mode, and the type of energy regeneration module is investigated, aiming at optimizing conditions for protein synthesis [12]. August Brookwell, Javin P. Oza, and Filippo Caschera instead compiled a review on CFPS, highlighting the rapid expansion of this platform technology, driven by its high adaptability to a broad range of production and testing schemes [13]. In particular, their analysis has covered topics such as the use of CFPS in metabolic engineering; “prototyping”; biosensing; biomanufacturing of monoclonal antibodies, antimicrobial peptides, small molecules, vaccines, and membrane proteins; and food biotechnologies. A section on the application of machine learning in CFPS is also present. Cheemeng Tan and colleagues proposed a quite original analysis of the innovation trend in CFPS [14]. A systematic study has been carried out on ~750 published patents and ~2000 peer-reviewed manuscripts in the field, reporting that the number of patent filings and published articles have strongly increased over the last decade and that geographical distribution of CFPS innovation is also quite dynamic. An increasing prevalence of biotechnology companies using cell-free systems has also emerged.

The Special Issue hosts two papers dealing with microcompartments—another key term in bottom-up SB. Confining biomolecular networks inside micrometric compartments such as lipid vesicles (or other types of compartments) leads to cell-like systems that have often been employed as models of current and primitive cells. Jian Xu, Tetsuya Yomo, and their collaborators have investigated, in a research article, the parameters affecting the efficient production of giant vesicles (GVs) made of phosphatidylcholines and phosphatidylethanolamines (with different hydrophobic chains) by using the water-in-oil inverted emulsions method [15]. The comprehensive comparison of yield, purity, size, stability, lipid composition, and encapsulation efficiency of the resulting GVs will be of great utility for further developments. The review of Ryo Mizuuchi and Norikazu Ichihashi deals the sustainable replication and evolution of genetic molecules such as RNA— which are essential requisites for the emergence of life [16]. A well-known problem that is inherent to these processes is the appearance of parasitic replicators. The Authors summarized the current understanding of the types and roles of primitive compartmentalization, especially from the perspective of the prevention of parasite replication. It is well known, in fact, that a possible mechanism to repress parasite amplification is compartmentalization, so that parasitic molecules are segregated, limiting their access to functional genetic molecules.

Two other reports complete our ensemble. The first one, co-authored by Chentao Yong and Andras Gyorgy, is an insightful numerical model of the system-level behavior of toggle switches in genetic circuits under conditions of limited availability of shared resources [17]. The Authors have revealed the details about how dynamic properties are affected in these conditions. The detrimental impacts of resource competition were demonstrated, as well as the exacerbation stemming from the unbalancedness of the switch. Finally, the team lead by Katarzyna P. Adamala highlights the history, function, and goals of the “Build-a-Cell” community, a global network of researchers that aims to develop synthetic living cells within the next decades [18]. The key role of large-scale collaboration is emphasized, as well as the fact that this principle has been made possible by Build-a-Cell’s open, collective structure.

It is evident that the collected articles largely focus on bottom-up SB approaches to synthetic cells (SCs). Because this is also our major area of expertise, we would like to take the opportunity to briefly comment on a couple of related issues.

The first is a consideration about the recent, rapid, and impressive growth of the research community interested in the subject. The studies on the construction of SCs are not so recent and date back to well before the 2000s because such approaches were already present in the origin-of-life research community [19,20]. Until the middle of the 2010s, only a limited number of groups were involved, yet the interest never fell. The contemporary flourishing developments somehow confirm the early visionary intuitions and foster this enterprise to new exciting levels. National and international consortia effectively boosted SC investigations to a prestigious level. Some examples are the Japanese Society for Cell Synthesis and the MaxSynBio, FabriCell, BaSyC, and SynCellEU initiatives, as well as the above-mentioned Build-a-Cell [18].

A second highlight refers, instead, to current trends. Without claiming to be fully comprehensive, we might mention some of the most interesting attempts of reconstituting cellular functions in a test tube or lipid vesicles (or other compartments), such as light-driven ATP synthesis [21], a partial cell division machinery [22,23], and self-reproduction machinery for translation factors [24,25], DNA [26,27], and ribosome [28,29]. These reconstituted systems reproduce the cellular functions in vitro to a certain extent, while the efficiency or yield are not comparable to the original ones in the cell. To fill in the gap would be one of the next important challenges.

Finally, we would like to comment on possible future directions. First is whole-SC self-reproduction. Now, researchers reconstitute various cellular functions ranging from DNA replication to translation, which should be combined as a total reproduction system. This challenge would require a different engineering technique from the previous reconstitution because it requires balancing all functions to work as a whole system. For that purpose, an evolutionary technique would be helpful [30], by which the most efficient artificial systems are autonomously selected. Second, because SCs have been often described with respect to fundamental questions in basic science (e.g., minimal requirements for life, non-life to life transition, experimental implementation of autopoietic systems), times are ripe to make further efforts and drive SC research also in applied science. In this respect, one can mention smart drug delivery agents [31], nanobioreactors, and biosensors.

In conclusion, the Special Issue articles confirm, without doubts, that SB in general, and bottom-up SC research in particular, are extremely lively and proficuous trends in current scientific investigations. While this Special Issue has been put forward for celebrating the past 10 years of *Life*, we wish it to also be a good omen for developing at to full extent the ‘Synthetic Biology and Systems Biology’ section.

## Data Availability

Not applicable.
